# The Effects of Cannabis on Female Reproductive Health Across the Life Course

**DOI:** 10.1089/can.2020.0065

**Published:** 2021-08-05

**Authors:** Daniel J. Corsi, Malia S.Q. Murphy, Jocelynn Cook

**Affiliations:** ^1^ Children's Hospital of Eastern Ontario Research Institute, Ottawa, Canada.; ^2^ Better Outcomes Registry & Network (BORN) Ontario, Ottawa, Canada.; ^3^ Clinical Epidemiology Program, Ottawa Hospital Research Institute, Ottawa, Canada.; ^4^ School of Epidemiology and Public Health, University of Ottawa, Ottawa, Canada.; ^5^ Department of Obstetrics and Gynecology, University of Ottawa, Ottawa, Canada.; ^6^ Society of Obstetricians and Gynaecologists of Canada, Ottawa, Canada.

**Keywords:** cannabis, marijuana, pregnancy, fertility, breastfeeding, menopause

## Abstract

**Introduction:** Cannabis is commonly used for its medicinal and therapeutic benefits and is also widely used as a recreational drug. Cannabis use has been increasing in Canada, including among Canadian women of reproductive age. Post-legalization, further increases in cannabis use are expected due to increased availability and lowered perceptions of harm. Although cannabinoids are well known for their effects on the central and peripheral nervous systems, endocannabinoid receptors have also been characterized throughout the female reproductive tract. Cannabinoids may affect many aspects of female reproductive health, including fertility, pregnancy outcomes with neonatal implications, and menopause.

**Purpose:** To provide a comprehensive review of trends in cannabis use among women and review the impact of cannabis across the female reproductive lifespan.

**Methods:** We searched PubMed and Cochrane Library databases using keywords and MeSH terms. Included studies reported the potential impact of cannabinoids on female fertility, pregnancy, transmission to breast milk, neonatal outcomes, and menopause.

**Results:** The existing literature is primarily concentrated on the effect of cannabis use in pregnancy and breastfeeding, with little exploration of its impact on fertility and in later life. Studies are limited in number, with small sample sizes, and are hampered by methodological challenges related to confounding and other potential biases.

**Conclusions:** There remain critical gaps in the literature about the potential risks of cannabis use, particularly in vulnerable populations, including pregnant women, women who are breastfeeding, and their infants. Given the rise in the prevalence of cannabis use, new, robust investigations into the consequences of cannabis exposure on female reproductive health are needed.

## Introduction

Cannabis is used widely for recreational, medicinal, and therapeutic purposes. Before legalization of cannabis for recreational sale and purchase in Canada, cannabis was the most widely used illicit drug,^1^ and its use was on the rise across all demographics and age groups, including among women of reproductive age.^1–3^

The primary psychoactive component of cannabis, tetrahydrocannabinol (THC), is rapidly absorbed by the bloodstream and spreads quickly throughout the body.^4^ Ingested and inhaled cannabinoids, including THC, cannabidiol and cannabinol, interact with receptors of the endogenous cannabinoid signaling system (ECSS), to produce wide-ranging effects on the central and peripheral nervous systems.^5,6^ The ECSS includes two primary G-protein-coupled receptors—CB1 and CB2—which are present throughout the body. Although primarily expressed in the brain and immune system, these endocannabinoid receptors have also been characterized throughout various tissues of the female reproductive tract.^5^

Exposure to cannabinoids may have differential impacts on female reproductive health across a woman's lifespan, from preconception to pregnancy, during breastfeeding, and during menopause. However, data on the potential short- and long-term health effects of cannabis use in women beyond of the perinatal window are infrequently and inconsistently described. In this review, we summarize available evidence on the impact of cannabis exposure across a woman's reproductive lifespan. We discuss the strengths and limitations of existing studies and highlight knowledge gaps for future research to address.

## Methods

A literature search was conducted in PubMed and Cochrane Library databases using keywords and MeSH terms for publications related to cannabis use and outcomes related to fertility, pregnancy, breastfeeding and menopause: cannabis, cannabinoids, cannabidiol, CBD, THC, marijuana, edible*, fertility, menstrual cycle, menses, menopause, pregnancy, pregnant, prenatal, perinatal, postnatal, breastfeed*, breastfed, lactation, nursing, fetus, fetal, neonatal, newborn, and child*.

We considered all study designs, including clinical trials, observational studies, systematic reviews and meta-analyses, clinical guidelines, and conference consensuses. Included studies were those reporting on data from female individuals, and cannabis as the intervention or exposure of interest. Animal studies were not the focus of this review. The year of publication, location, and environment of published studies were not limited. The reference lists of included articles were screened to identify publications that may have been missed by the search strategy.

## Cannabis and Female Fertility

Endocrinology studies in animals have consistently shown that the female reproductive system is sensitive to the effects of cannabinoids. CB1 and CB2 type endocannabinoid receptors for THC are expressed in the oviduct, uterus, and anterior pituitary.^7^ Knockout models for CB1 and CB2 receptors exhibit defects in fertilization and implantation.^8–10^ Experimental studies on rodents and nonhuman primates have demonstrated reductions in sex hormone levels and ovulatory disruption following THC administration.^11–15^ Although data on the impact of cannabis use on human fertility are few, available evidence suggests that cannabinoid exposure does indeed have measurable impacts on female reproductive function (Table 1).

**Table 1.** References Reporting on Cannabis Exposure and Female Fertility

Reference and setting	Study design	No. female participants	Measure of cannabis exposure	Exposure window	Endpoints evaluated	Observed effect of cannabis exposure
Mendelson et al.^77^ USA	Interventional study	*N*=8. Healthy adult females in the periovulatory phase of their menstrual cycles	1-g cannabis containing 1.8% THC	Acute administration. Controlled cannabis smoking for 12 min.	Plasma LH, prolactin, estradiol, progesterone at 15, 20, 25, 30, 45, 60, 90, 120, 150, 180 min after smoking initiation	↑ LH levels ↑ Prolactin levels
Mendelson et al.^21^ USA	Interventional study	*N*=16. Healthy adult females in the follicular (*n*=8) and luteal phases (*n*=8) of their menstrual cycles	1-g cannabis containing 1.8% THC versus placebo cigarette	Acute administration. Controlled cannabis smoking for 10–12 min	Plasma LH at 15, 20, 25, 30, 45, 60, 90, 120, 150, 180 min after smoking initiation	↓ LH levels during luteal phase
Mueller et al.^25^ USA	Cross-sectional study	*N*=300. Women unsuccessfully trying to conceive for at least 12 months (*n*=150), matched to women who had recently given birth (*n*=150).	Self-report	Lifetime	Primary ovulatory infertility Primary tubal infertility	↑ Risk of primary ovulatory infertility
Block et al.^19^ USA	Cross-sectional study	*N*=56	Self-report	Previous 3 months	Serum testosterone, prolactin, FSH, LH, cortisol	No effect
Jukic et al.^16^ USA	Prospective cohort study	*N*=217. Women planning pregnancy	Self-report	Previous 2 years	Follicular phase length	↑ Follicular phase length
White et al.^18^ USA	Cross-sectional study	*N*=913. Premenopausal women	Self-report	Lifetime	Serum AMH	No effect
Slavin et al.^22^ USA	Cross-sectional study	*N*=145. Women experiencing PMS and PMDD who had used cannabis at least once in their lifetime	Self-report	Previous 1 year	Expectancy of cannabis-induced relief from PMS and PMDD symptoms	Expectancies of cannabis-induced relief from PMS/PMDD symptoms positively correlated with monthly cannabis use and severity of PMS/PMDD symptoms.
Lammert et al.^17^ USA	Prospective cohort study	*N*=52. Women who co-used cannabis and tobacco, matched to women used only tobacco	Self-report	“Current use”	Evidence of LH surge Follicular phase length Luteal phase length Total menstrual cycle length	↓ Luteal phase length
Kasman et al.^23^ USA	Cross-sectional study	*N*=1076. Women actively trying to conceive	Self-report	Previous 12 months	Time to pregnancy	No effect
Wise et al.^24^ USA and Canada	Prospective cohort study	*N*=4194. Women with ≤6 cycles of pregnancy attempts time at study enrolment	Self-report	Previous 2 months	Fecundability. Data assessed every 8 weeks over 12 months or until pregnancy, initiation of fertility treatment or loss to follow-up	No effect

AMH, anti-Mullerian hormone; FSH, follicle stimulating hormone; LH, luteinizing hormone; PMDD premenstrual dysphoric disorder; PMS, premenstrual syndrome; THC, tetrahydrocannabinol.

### Ovulation

Cannabis may affect sex hormones essential to fertility and the timing of ovulation. In a sample of 217 women planning pregnancy, Jukic et al. relied on self-reported cannabis use frequency (number of times smoking cannabis in the past month) and urine estradiol and progesterone levels to determine the effect of cannabis exposure on the timing of ovulation.^16^ Follicular phases were 3.5 days longer among individuals reporting occasional cannabis use (1–3 times in the past 3 months, *p*=0.04), and 1.7 days longer among frequent cannabis users (>3 times in the past 3 months, *p*=0.04 compared to non-users). Cannabis users also had more anovulatory cycles compared with nonusers (43% vs. 15%), from which the authors inferred potential ovulatory delay and cycle inhibition associated with cannabis use.

Lammert et al. compared the menstrual cycles of premenopausal women reporting co-use of tobacco and cannabis (*n*=13) with age-matched controls who used tobacco only (*n*=39).^17^ The luteal phases of women who reported co-use of tobacco and cannabis were 5.4 days shorter than for women who used tobacco only (mean 11.4 days±SD 2.2 vs. 16.8 days ±11.3, *p*=0.002). In this study, there was no apparent association between luteinizing hormone (LH) surge suppression and cannabis use, although cannabis use was based on self-report (yes/no), and no additional information on timing or frequency of use was collected.

In a cross-sectional analysis of 913 women, White et al. found no association between past or current cannabis use and serum anti-Müllerian hormone (AMH) levels.^18^ AMH is an indirect marker of fertility and ovarian follicular reserve, and correlates with IVF success rates and time to menopause. Cannabis smoking status, use frequency, years of use, and age of initiation were not associated with serum AMH levels. There were very few current cannabis users in this study (<1%), however, limiting the ability to draw conclusion from their findings. Another study of 39 nonusers and 17 chronic cannabis users reporting at least weekly cannabis use, found that past 3-month cannabis use did not affect serum testosterone, cortisol, LH, follicle stimulating hormone (FSH), or prolactin^19^; stratification by frequency of cannabis use did not change the study findings.

The acute effects of cannabis smoking on sex hormones in reproductive-aged women have also been examined. In one of the earliest clinical studies on the topic, Mendelson et al. performed time-course experiments in small groups of healthy female volunteers with self-reported “normal menstrual cycles” and no use of birth control medication or intrauterine devices to evaluate the effect of smoked cannabis on sex hormones. In the first such experiment, eight periovulatory participants each consumed a 1-g cannabis cigarette containing 1.8% THC and completed sex hormone testing at 15, 20, 25, 30, 45, 60, 90, 120, 150, and 180 min after smoking initiation. Cannabis smoking induced a statistically significant increase in LH and prolactin levels 20 min after smoking initiation (*p*<0.01 for both).^20^ There were no changes in estradiol or progesterone levels. Using a similar experimental design, the same authors assessed the effect of cannabis smoking on female sex hormones during the luteal phases of 16 women, eight of whom were administered a cannabis cigarette and eight who were administered a placebo cigarette.^21^ Cannabis smoking induced a 30% suppression in LH levels from 60 to 120 min after administration (*p*<0.02). At the end of the 180 time-course experiment, LH levels were lower after cannabis administration than placebo (*p*<0.04). No changes in estradiol or progesterone levels after cannabis smoking were observed.

Cannabis is also used to alleviate the signs and symptoms of premenstrual syndrome (PMS) and premenstrual dysphoric disorder (PMDD), although there have been few formal investigations in this area. Data suggest that the severity of PMS/PMDD is correlated with positive expectations for cannabis to ameliorate PMS/PMDD symptoms, such as irritability and joint and muscle discomfort, and also use frequency.^22^ With the wider availability of cannabis as many jurisdictions move toward legalization of the drug, more work is required to evaluate the efficacy of cannabinoid products on treating PMS symptoms, particularly in light of the potential repercussions on fertility.

### Time to conception

There are limited data on the impact of cannabis use on time to conception. Kasman et al. assessed the impact of cannabis use on fertility in a population-based sample of 1076 women who were actively trying to become pregnant.^23^ Using a current duration approach to assess fecundity, the authors relied on survey responses to the question, “How long have you been trying to become pregnant.” A total of 124 (12.5%) women reported cannabis smoking in the preceding 12 months while attempting to conceive. There were no observed effects of cannabis use on time to pregnancy; adjusted time ratio of 1.03 months (95% confidence interval [95% CI] 0.80–1.31) for nonusers versus any users. Cannabis use frequency (monthly, weekly, daily) did not affect time to pregnancy, and no significant interactions with respondent age, race, income, marital status, parity, and infertility care.

In contrast, Wise et al. used data from a web-based preconception cohort study to evaluate the association between use of cannabis and fecundability (per cycle probability of conception).^24^ The authors reported no association between cannabis use and fecundability. Compared with nonusers, female respondents reporting cannabis use in the previous 2 months had fecundability ratios of 0.99 (95% CI 0.85–1.16) for cannabis use <1 time per week and 0.98 (95% CI 0.80–1.20) for cannabis use at least once per week. In a cohort of 300 women seeking evaluation for infertility challenges, Mueller et al. demonstrated that cannabis use history was associated with an increased risk for primary ovulatory infertility compared with women who had never used cannabis (relative risk [RR] 1.77, 95% CI 1.0–3.0).^25^ The risk for infertility was greatest among women who had used cannabis within 1 year of trying to become pregnant (RR 2.1 95% CI 1.1–4.0).

Increases in cannabis use are greatest among individuals of reproductive age.^1,26^ Further increases in use prevalence are anticipated as a result of increased availability and lowered perceptions of harm associated with cannabis legalization. The lack of contemporary data on the potential consequences of cannabis exposure to female fertility highlights a need for a more substantive investigation in this area.

## Cannabis Use in Pregnancy

In pregnancy, cannabinoids and their metabolic byproducts cross the placenta, enter the fetal bloodstream, and distribute to fetal tissues, including the brain.^6^ Non-human studies show that THC reaches fetal plasma within 15 min of maternal exposure, and equilibrates to maternal levels within 3 h.^6^ The lipophilic nature of THC, together with a half-life of up to 8 days in fatty tissues, results in its slow clearance from fetal tissues. Fetal exposure is, therefore, prolonged even after maternal discontinuation.^27,28^ The number of different cannabis product formats are rapidly increasing with the production of vaporizers, tinctures, novel consumables, extracts, and oils, each of which may have different risk implications. Robust evidence about safety and metabolism of these forms is currently lacking.

Determining the consequences of perinatal cannabis exposure is challenging due to difficulties in differentiating exposed from unexposed children. Self-report is the most widely used method to evaluate substance use during pregnancy, including cannabis.^29^ Although economical and reasonably valid for epidemiological studies,^30^ self-reports can suffer from bias and measurement error. Misreporting or inaccuracies in reporting cannabis use in pregnancy may arise related to feelings of guilt; fear of legal action, later child apprehension, or social stigmatization; poor recall of drug-use details; poorly constructed screening tools; or inadequate training of interview staff.^30–32^ Biospecimen analysis offers a more objective strategy, and can also alleviate challenges related to unmeasured second-hand or co-exposure.^33–35^ Biospecimen collection, storage, and analysis are costly, however, and there have been few epidemiological studies in pregnancy where cannabis use has been confirmed by biospecimen analysis.

### Prevalence of cannabis use in pregnancy

Rates of cannabis consumption are increasing among women of reproductive age, those who are pregnant, and those who are breastfeeding. In Canada, pre-legalization data from the province of Ontario demonstrate that cannabis use in pregnancy increased from 1.2% in 2012 to 1.8% in 2017; a relative increase of 61%.^2^ Similar increases have been observed in the Canadian province of British Columbia and in other countries, where available data suggest that peripartum cannabis use based on self-reports and toxicology varies from 1% to 8%.^3,30,36–40^ Around 3.9% of the 4971 pregnant women who participated in the *U.S. National Survey on Drug Use and Health* reported last-month cannabis use. Use prevalence was highest at 7.4% in the first trimester of pregnancy.^39^ The National Birth Defect Prevention Study, also based in the United States, found that 4% of the 15,208 mothers confirmed cannabis use in the periconceptional period.^41^

In an international multicenter study of nulliparous women (*n*=5888), self-reported cannabis use between 15 and 20 weeks' gestation was 3.9%.^37^ In France, a substance use survey of 13,545 mothers, who had delivered 2–3 days earlier, found that self-reported cannabis use rate was 1.2% in pregnancy.^36^ In the Netherlands, 214 (2.9%) of the 7452 women participating in the Generation R prospective cohort study reported cannabis use before and during the first trimester of pregnancy. A total of 41 (19%) of cannabis users in this cohort continued to use throughout pregnancy.^42^ Finally, a retrospective cohort study (*n*=24,874) conducted in Australia, found that the prevalence of lifetime cannabis use was 9.5% for Australian women and 2.6% in pregnancy.^38^

### Associations with perinatal outcomes

Cannabis exposure during pregnancy has been implicated in a wide range of adverse perinatal outcomes (Table 2), including stillbirth,^3,43^ preterm birth,^3,37,38,44^ low birth weights^38,42,45^ and other measures of fetal/infant growth,^46–48^ small for gestational age,^37,38,44^ and increased admission to the neonatal intensive care unit (NICU).^38,44,47^ A 2016 meta-analysis of 24 case–control, cross-sectional and cohort studies indicated that women who reported cannabis use during their pregnancy had higher odds of being anemic (pooled odds ratio [pOR] 1.36, 95% CI 1.10–1.69) compared with nonusers, and their infants more likely to have lower birth weight (pOR 1.77, 95% CI 1.04–3.01) and require transfer to NICU (pOR 2.02, 95% CI 1.27–3.21).^47^ With loosening restrictions on cannabis use in many jurisdictions, new data in this area are constantly emerging; and the field will soon warrant a contemporary systematic review and meta-analysis.

**Table 2.** References Reporting on Cannabis Exposure and Perinatal Outcomes

Reference and setting	Study design	No. female participants	Measure of cannabis exposure	Exposure window	Endpoints evaluated	Observed effect of cannabis exposure
Tennes et al.^53^ USA	Prospective cohort study	*N*=756	Self-report	During pregnancy (overall and by trimester)	Infant sex, length, head circumference, palpebral fissures, muscle tone, physical anomalies, birth weight, gestational age, Apgar scores, neonatal complications	↑ Proportion of male infants ↓ Infant length ↑ Gestational age
Day et al.^46^ USA	Prospective cohort study	*N*=519	Self-report	Before pregnancy During pregnancy (by trimester)	Newborn length, head and chest circumference, Apgar scores, birth weight, gestational age, Ponderal index, congenital abnormalities	Cannabis exposure in first trimester associated with: ↓ Newborn length ↑ Ponderal index
Fergusson et al.^48^ United Kingdom	Prospective cohort study	*N*=12,129	Self-report	6 month before pregnancy and up to 20 weeks gestation	Late fetal and perinatal death Special care admission Birth weight, length, and head circumference	↓ Birth weight (no effect after adjusting for confounders)
El Marroun et al.^42^ Netherlands	Prospective cohort study	*N*=7452	Self-report	Before pregnancy During pregnancy (in the 3 months before survey administration. Timing of administration not limited)	Birth weight and fetal growth measures in early, mid and late-pregnancy (femur length, abdominal and head circumference, transcerebellar diameter, estimated fetal weight)	Cannabis exposure in early pregnancy associated with: ↓ Fetal head circumference and estimated fetal weight in mid- and late pregnancy; ↓ birth weight Continued cannabis use throughout pregnancy associated with: ↓ Estimated fetal weight in mid- and late pregnancy; ↓ Birth weight No effect of cannabis use before pregnancy on fetal growth in mid- and late pregnancy or at birth
Hayatbakhsh et al.^38^ Australia	Retrospective cohort study	*N*=24,874	Self-report	Lifetime During Pregnancy (history taken at 12–16 weeks gestation)	LBW PTB Birth length Apgar score at 5 min SGA NICU admission	↑ Risk of LBW ↑ Risk of PTB ↑ Risk of SGA ↑ Risk of NICU admission
Saurel-Cubizolles et al.^36^ France	Cross-sectional study	*N*=13 545	Self-report	During pregnancy	PTB (spontaneous and medically indicated) SGA	↑ Risk of spontaneous PTB
Varner et al.^43^ United States	Cross-sectional study	*N*=2595	Self-reported “lifetime drug use” and Carboxy-THC testing at delivery (umbilical cord tissue, maternal serum)	Lifetime (by self-report) Near delivery (for biological sample testing)	Stillbirth	↑ Risk of stillbirth
Leemaqz et al.^37^ Australia, New Zealand, Ireland, United Kingdom	Prospective cohort study	*N*=5588	Self-report	During pregnancy, in the 3 months before survey administration at 15 and 20 weeks gestation	Spontaneous PTB, SGA, pre-eclampsia, gestational diabetes mellitus	Continued cannabis use at 20 weeks' gestation associated with: ↑ Risk of spontaneous PTB
Gunn et al.^47^ International	Systematic Review and meta-analysis	24 studies	Combination of self-report and biological testing	During pregnancy	Maternal, fetal, and neonatal outcomes up to 6 weeks postpartum	↑ Risk of maternal anemia ↑ Risk of infant LBW ↑ Risk of infant transfer to NICU
Corsi et al.^2^ Canada	Retrospective cohort study	*N*=661,617	Self-report	During pregnancy	Perinatal outcomes: PTB, SGA, placental abruption, stillbirth Maternal outcomes: pre-eclampsia, gestational diabetes, mode of delivery Neonatal outcomes; transfer to NICU, 5 min Apgar <4	↑ Risk of PTB ↑ risk of SGA (<3rd percentile) ↑ Risk of placental abruption ↑ Risk of transfer to NICU ↑ Risk of 5 min Apgar <4
Luke et al.^3^ Canada	Retrospective cohort study	*N*=243 140	Self-report	During pregnancy, before the first prenatal visit	Stillbirth (antepartum intrapartum), SGA, spontaneous PTB	↑ Risk of SGA ↑ Risk of spontaneous PTB ↑ Increased risk of intrapartum stillbirth
Gabrhelík et al.^45^ Norway	Prospective cohort study	*N*=10,101 pregnancies	Self-report	Use earlier in life Use last month before pregnancy During pregnancy (before week 17/18; between weeks 17/18 and 30; after week 30) After pregnancy (to 6 months postpartum)	Birth weight, birth length, head circumference, PTB, malformations, Apgar at 1 min, 5 min, SGA, gestational length	Prolonged cannabis use associated with (use during 2 or more of the study periods): ↓ Birth weight

LBW, low birth weight; NICU neonatal intensive care unit; PTB, preterm birth; SGA, small for gestational age.

### Associations with longer-term child outcomes

Fetal and infant exposure to cannabis during pregnancy and breastfeeding can disrupt the fetal ECSS, which is present and active from the early embryonic stage and modulates neurodevelopment into adulthood.^6^ Perturbations to this system have a range of cellular effects and may affect cognitive, behavioral, and emotional development in childhood.^6,49,50^ Several longitudinal studies have reported on the association between prenatal cannabis exposure and childhood neurodevelopmental outcomes.^51–53^

Tennes et al. assessed weight, height, and psychomotor outcomes in 129 infants, 1 year after delivery.^53^ Thirty-eight (29.5%) infants were born to mothers who self-reported heavy cannabis use (once or more daily), and 44 (34.1%) were born to mothers with light or moderate (one time only to once a week; more than once a week but less than daily) cannabis use during pregnancy. The authors found no measurable differences between exposed and unexposed infants. The Ottawa Prenatal Prospective Study followed children born from 1980–1983 to 250 predominantly healthy, Caucasian, middle-class women in Ottawa, Canada. The study included 200 children whose mothers reported using cannabis, alcohol, or tobacco during pregnancy and a comparison group of 50 nonusers.^54–57^ Based on maternal self-report, 47 children had documented prenatal cannabis exposure, averaging about 1 joint per week during pregnancy. Exposed neonates had increased startle response, tremors, and deficient habituation to visual stimuli versus unexposed neonates,^54^ and at 4 years of age had lower scores on verbal and memory domains of McCarthy Scales of Children's Ability.^55^ No effects were found at ages 5–6, 6–9, 9–12, or 13–16 years after adjusting for home environments.^56–58^

The Maternal Health Practices and Child Development Study (Pittsburgh; initiated in 1982), interviewed 1360 randomly selected women from an inner-city outpatient prenatal clinic.^46^ Participants were predominantly unmarried, low-income, and 50% were African American. The authors followed women who self-reported >2 joints per month in the first trimester, and a random sample of women who used “less than this amount” as controls.^46^ The authors reported adverse effects of cannabis exposure on child performance on the Stanford–Binet Intelligence Scale at 3 years of age. Effects were limited to exposure during the first and second trimesters.^59^ Second trimester exposure was also associated with increased impulsivity, hyperactivity, and delinquency; and decreased concentration, IQ score, and verbal and visual reasoning at 6–10 years of age.^60^

The Generation R study (Rotterdam, Netherlands; initiated in 2002) also relied on self-report to collect data on cannabis use in pregnancy.^61^ Among exposed children (88 of 4077), only girls exhibited significant increases in attention problems at 18 months of age based on the Child Behavior Checklist (OR 2.75, 95% CI 1.27–5.96).^61^ Aggressive behavior was also increased among girls at this age, but the association was not statistically significant (OR 1.66, 95% CI 0.38–7.26). The Adolescent Brain Cognitive Development study (multisite, USA; initiated in 2005).^62^ Of 4361 children, 201 (4.6%) had prenatal cannabis exposure based on maternal self-report. Fifty-six had prenatal exposure after maternal knowledge of pregnancy.^52^ Follow-up of these children at 9–11 years of age revealed small increases in risk for psychotic-like experiences (unstandardized β 1.41, 95% CI 0.34–2.48).

## Cannabis and Breastfeeding

The low molecular weight and high lipid solubility of cannabinoids contribute to its propensity for transfer into breast milk.^4,63–65^ The pharmacokinetics of cannabinoids in breast milk are not well understood; however, the transfer of cannabinoids to maternal breast milk is likely subject to maternal dosing and frequency of dosing. The variable fat composition of human milk and the milk sample type (hindmilk has a substantially higher lipid content than foremilk)^66,67^ can further affect the cannabinoid content of a given sample. In a field where determining cannabis exposure is still largely reliant on self-reported data that do not include comprehensive use profiles, it is unsurprising that cannabis and breast milk data are lacking.

### Transfer to breast milk and the neonate

Case reports confirming the presence of cannabinoids in the breast milk of mothers who consume cannabis during lactation^63,68^ have prompted the development and validation of new cannabinoid detection methods in this matrix,^69^ and investigation into its pharmacokinetic properties.^65,70^ In a 1982 correspondence published in the New England Journal of Medicine, Perez-Reyes and Wall reported the presence of cannabinoids in the breast milk of two chronic cannabis users.^68^ Cannabinoid concentrations in breast milk were eight times higher than in maternal serum concentrations. Fecal samples from one of the infants were also positive for THC and its principal metabolites 11-hydroxy-THC and 9-carboxy-THC.

More recently, Crume et al. investigated the prevalence of postpartum maternal cannabis use in a population-based sample of 3285 mothers responding to the 2014–2015 Colorado Pregnancy Risk Assessment Monitoring System survey.^71^ The prevalence of postnatal cannabis use in this cohort was 5% (95% CI 4.1–6.2), and 10.2% (95% CI 7.1–14.6) among women who breastfed in the first 8 weeks after delivery. Prenatal and postnatal cannabis use was associated with a shorter duration of breastfeeding. Bertrand et al. used samples from a human milk biorepository to quantify cannabinoids in the samples of mothers who had reported cannabis use within 14 days of milk sample collection.^65^ Cannabinoids were detectable in 34 (63%) of the 54 analyzed samples, up to 6 days after the last reported use. The frequency of cannabis use and time between consumption and sample collection were significant predictors of THC milk concentrations.

Finally, in a pilot pharmacokinetic study, Baker et al. evaluated the transfer of THC metabolites into the breast milk of eight volunteers after a known THC dose.^70^ Participants were 18–45 years of age, a median 5 months postpartum (range 3–5 months), and all reported smoking cannabis while exclusively breastfeeding. Participants were instructed to purchase a preweighed 0.1 g sample of cannabis with 23.18% THC content from a prespecified dispensary. Samples were smoked after 24 h of abstinence from cannabis use, and milk samples collected before smoking, and 20 min, 1 h, 2 h, and 4 h after inhalation. THC concentrations in maternal milk peaked at 1 h (mean 94 ng/mL, range 21.2–420.3 ng/mL) and declined slowly over the remaining study period.

### Cannabis exposure through breast milk and infant outcomes

Distinguishing the effects of prenatal and postnatal exposures is challenging as women are unlikely to begin using cannabis *de novo* in the postpartum period after abstaining in the periconceptional and gestational periods. It is, therefore, unsurprising to find limited data on health outcomes of infants exposed to cannabis through breast milk (Table 3). Astley and Little evaluated motor and mental development at 1 year of age among 68 breastfed infants whose mothers reported cannabis use during lactation compared with 68 matched controls without lactational cannabis exposure. Infant exposure to cannabis through breast milk in the first month postpartum was associated with an average 14-point decrease in the Bayley index of infant motor development after adjusting for maternal smoking, drinking, and cocaine use during pregnancy and lactation. However, both cases and controls had prenatal cannabis exposure, making it difficult to delineate the effects of prenatal and postnatal cannabis exposure on the study outcome measures.^72^ Tennes et al. also used the Bayley Infant Scale of Mental and Motor Development to assess infant outcomes following prenatal and postnatal cannabis exposure. In a cohort with a high proportion of cannabis users, 62 infants were breastfed and 27 (43.5%) mothers reported using cannabis during breastfeeding.^53^ The authors found no differences in infant height, weight, or psychomotor development. Age of infant weaning was also similar between the two groups, suggesting that cannabis use did not interfere with lactation.

**Table 3.** References Reporting on Cannabis Exposure from Breast Milk and Neonatal Outcomes

Reference and setting	Study design	No. infant participants	Measure of cannabis	Exposure window	Endpoints evaluated	Observed effect of cannabis exposure
Tennes et al.^53^ USA	Prospective cohort study	*N*=62	Self-reported	Within 1 year postpartum or during lactation	Weight, height, age of weaning, Bayley Infant Scale scores at 1 year (PDI, MDI)	No effect
Astley and Little^72^ USA	Retrospective cohort study	*N*=136. Mother–infant dyads with cannabis exposure during lactation (*n*=68) matched to dyads without exposure during lactation (*n*=68). All dyads, had prenatal cannabis exposure.	Self-report Confirmed by unspecified drug tests	1–3 months postpartum, or during lactation	Bayley Infant Scale scores at 1 year (PDI, MDI)	↓ Infant motor development associated with cannabis exposure through breast milk during the first month postpartum

MDI, mental developmental index; PDI, psychomotor developmental index.

### Clinical recommendations

In the absence of robust and contemporary data, the Society for Obstetricians and Gynecologists of Canada^73^ and the American College of Obstetricians and Gynecologists,^74^ both encourage abstinence from cannabis during lactation. The guidelines do not, however, recommend against breastfeeding while using cannabis. Women who use cannabis and breastfeed should be supported to breastfeed well, and this can be supported, for example, through referral to a lactation consultant.

## Cannabis and Menopause

Cannabis use is frequently cited as a way to mitigate health concerns that are common during the perimenopausal period.^75^ Symptoms associated with menopause include hot flashes, irritability, depressed moods, poor sleep, joint and muscle pain, vaginal dryness, and urinary symptoms. The duration of the menopausal transition varies between individuals but typically lasts 2–6 years.^76^ Treatment options for symptoms include hormone replacement therapy, antidepressant medications, and herbal and other alternative therapies. Data reporting on observations in perimenopausal women are summarized in Table 4.

**Table 4.** References Reporting on Cannabis Exposure and Menopause

Reference and setting	Study design	No. female participants	Measure of cannabis exposure	Exposure window	Endpoints evaluated	Observed effect of cannabis exposure
Mendelson et al.^77^ USA	Interventional crossover study	*N*=10 menopausal women who were naive cannabis users	1-g cigarette containing 1.83% THC versus placebo	Acute administration on 2 occasions, 11 days apart. Controlled cannabis smoking for 15 min	Plasma LH, pulse rate and self-assessed intoxication at 15, 20, 25, 30, 45, 60 90, 120, 150, 180 min after initiation of smoking	No effect on LH ↑ Pulse rate ↑ Level of intoxication
Slavin et al.^75^ USA	Interventional crossover study	*N*=115 menopausal and postmenopausal women who had used cannabis at least once in their lifetime	Self-reported	Previous 1 year	Expectancy of cannabis-induced relief from menopausal symptoms	Symptoms were positively correlated with cannabis use frequency and expectancies of cannabis-induced relief from menopause symptoms. Expectancies of cannabis-induced relief from symptoms also correlated with frequency of use and menopause symptoms.

Mendelson et al. evaluated the acute effects of cannabis smoking in 10 healthy menopausal volunteers with naive cannabis use histories in a small double-blinded crossover study.^77^ Cannabis smoking (acute administration of a 1-g cigarette with 1.83% THC) did not elicit changes in plasma LH levels compared with a placebo cigarette.^77^ The effects of cannabis smoking on menopausal symptoms were not evaluated. More recently, Slavin et al. surveyed 115 menopausal and postmenopausal women with favorable perceptions toward cannabis use to evaluate expectancy-mediated effects of cannabis on menopausal symptoms.^75^ Frequency of self-reported cannabis use was significantly correlated to the number and severity of menopausal symptoms, as was the expectancy of cannabis-induced symptom amelioration. Cannabis was not perceived to be equally effective for all symptoms, however. Whereas cannabis was perceived to be largely beneficial for treating joint/muscle discomfort, irritability, sleep problems, depression, anxiety, and hot flashes, this was not the case for other symptoms such as heart discomfort, exhaustion, vaginal dryness, and bladder problems.

In summary, although some users may find cannabis to be beneficial for ameliorating signs and symptoms commonly associated with menopause women (e.g., insomnia, irritability, join pain, depression),^75^ there are few data on the efficacy and safety of cannabis use in this context. Given that positive expectancies of cannabis-induced relief may influence the frequency and quantity of cannabis use, further research is warranted to ensure that consumers can make decisions in line with supporting evidence.

## Summary

Approximately 11% of women in Canada report cannabis use, and use rates are higher (18%) among women 15–49 years of age.^78^ The literature indicates that cannabis exposure has health implications for women, the effects of which vary across the life course. This review summarized the effects of cannabis exposure on female health from fertility, pregnancy and neonatal outcomes, breastfeeding, and menopause.

Chronic cannabis use may reduce female fertility, although few studies exist in this area. Cannabis use in pregnancy is associated with adverse neonatal outcomes, particularly, low birth weight, preterm birth, admission to neonatal intensive care, and small for gestational age. Inconsistencies in reported associations between cannabis use and perinatal outcomes may be related to statistical adjustment for confounding factors, exposure ascertainment, other significant differences between women who use cannabis and those who do not, and the inherent limitations of observational study designs. International studies have also shown that women who use cannabis are more likely to be younger; unmarried; have lower income and education; and use alcohol, tobacco, and other illicit substances. We found evidence that cannabis and its metabolites can transfer to breast milk. There are currently limited data on the potential effects of cannabis exposure through breast milk on infants. Finally, studies on cannabis and menopause indicate that cannabis is not associated with plasma LH levels but may be used to some benefit to alleviate specific symptoms of menopause.

Cannabis use is increasing, and this is concurrent with raised social acceptability and lowered perceptions of harm. There remain critical gaps in the literature about the potential risks of cannabis use on female fertility, during pregnancy, breastfeeding, and on the longer-term outcomes of exposed infants.

## Abbreviations Used

95% CI: 95% confidence interval
AMH: anti-Mullerian hormone
ECSS: endogenous cannabinoid-signaling system
FSH: follicle stimulating hormone
LBW: low birth weight
LH: luteinizing hormone
MDI: mental developmental index
NICU: neonatal intensive care unit
PDI: psychomotor developmental index
PMDD: premenstrual dysphoric disorder
PMS: premenstrual syndrome
pOR: pooled odds ratio
PTB: preterm birth
RR: relative risk
SD: standard deviation
SGA: small for gestational age
THC: tetrahydrocannabinol
